# Seasonal Variation of a New Brazilian Greenish-Brown Propolis Type: Chemical Composition and Antioxidant, Antimicrobial, and Antileishmanial Activities

**DOI:** 10.3390/molecules31091447

**Published:** 2026-04-27

**Authors:** Emanoel Guilhermino da Silva, Arthur Luy T. Ferreira Borges, João Victor L. de Oliveria, Rodrigo J. Nunes Calumby, Salvana P. Manso Costa, Pierre Barnabé Escodro, Isabel Cristina Celerino de Moraes Porto, Ana Paula do Nascimento Prata Lins, Maria Aline B. Fidelis de Moura, Camila B. Dornelas, Johnnatan Duarte de Freitas, Regianne U. Kamiya, Lara Mendes Almeida, Louisianny Guerra da Rocha, Edmilson Rodrigues da Rocha Junior, Marília O. F. Goulart, Ticiano Gomes do Nascimento

**Affiliations:** 1 Postgraduate Program of Pharmaceutical Science, Institute of Pharmaceutical Science, Federal University of Alagoas, Maceio 57072-970, Alagoas, Brazilarthurltfb@gmail.com (A.L.T.F.B.); joao.oliveira@esenfar.ufal.br (J.V.L.d.O.); rjnc_biomed@hotmail.com (R.J.N.C.); salvanacosta@gmail.com (S.P.M.C.); aline.fidelis@icf.ufal.br (M.A.B.F.d.M.); camila.dornelas@icf.ufal.br (C.B.D.); ruk@icbs.ufal.br (R.U.K.); larameal@gmail.com (L.M.A.); 2 Postgraduate Program of Material Science, Center of Technology, Federal University of Alagoas, Maceio 57072-970, Alagoas, Brazil; johnnatan.duarte@ifal.edu.br; 3 Postgraduate Program of Biological and Health Sciences, ICBS, Federal University of Alagoas, Maceio 57072-970, Alagoas, Brazil; 4 Postgraduate Program of Veterinary Science, CECA, Federal University of Alagoas, Viçosa 57700-000, Alagoas, Brazil; pierre.escodro@vicosa.ufal.br; 5 Postgraduate Program of Dentistry, FOUFAL, Federal University of Alagoas, Maceio 57072-970, Alagoas, Brazil; 6 Postgraduate Program of Plants Protection, CECA, Federal University of Alagoas, Rio Largo 57100-000, Alagoas, Brazil; ana.prata@ceca.ufal.br; 7 Laboratory of Instrumental Chemistry, Department of Chemistry, Federal Institute of Alagoas, Maceio 57065-660, Alagoas, Brazil; 8 Laboratory of Parasitology, Centre of Biosciences, Federal University of Rio Grande do Norte, Lagoa Nova, Natal 59078-970, Rio Grande do Norte, Brazil; louisianny@yahoo.com.br; 9 Postgraduate Program of Chemistry and Biotechnology, Institute of Chemistry and Biotechnology, Federal University of Alagoas, Maceio 57072-900, Alagoas, Brazil; edmilsonrrj@gmail.com (E.R.d.R.J.); mofg@qui.ufal.br (M.O.F.G.)

**Keywords:** propolis raw material, flavanones, hydroxycinnamic acids, chromatographic profile, antioxidant activity, antibacterial and antileishmanial activities, multivariate analysis

## Abstract

Propolis is a natural product of honey bees whose chemical composition is influenced by different plant species and environmental factors, resulting in diverse biological activities. A new propolis type, the greenish-brown propolis (GBPUP), was identified in the northeast of Brazil. This study aimed to evaluate the influence of seasonal variation in the chemical composition of GBPUP extracts over a 12-month period. LC–ESI–Orbitrap–FTMS and UFLC–DAD–UV–Vis revealed a chemical composition with some differences to that of Brazilian green propolis, with pinocembrin as the major compound, followed by galangin, pinostrobin, chrysin, artepillin C, and pinobanksin. The extracts exhibited high levels of total phenolic, flavonoid, and flavanone contents and moderate to high antioxidant activity. Circos plot analysis showed that specific metabolites were responsible for the high activity against *S. aureus* (artepillin C, kaempferol, and ferulic acid) and *C. albicans* (galangin, pinobanksin, chrysin, and pinocembrin) and for moderate antibacterial activity against *E. faecalis* (rutin) and *E. coli* (luteolin, rutin, quercetin, and caffeic acid). ANOVA simultaneous component analysis (ASCA) showed a strong correlation between the metabolites (*p*-coumaric acid, artepillin C, luteolin) and leishmanicidal activity. Thus, seasonal evaluation allowed the identification of bioactive molecules, the months with greater bioactivity of the GBPUP extracts representing the first comprehensive study of the seasonality of this new and promising propolis variety.

## 1. Introduction

Propolis, also called “bee glue”, is a sticky, viscous [1] natural chemical mixture with a characteristic odor [2], accumulated by honey bees after mixing their wax and salivary enzymes with pollen and plant resin [3]. Resins can be obtained from different plant parts, such as the exudate of flowers, leaves and buds [4].

Propolis has anti-inflammatory, antiviral, antibacterial [5], antiparasitic [6], astringent, phyto-inhibitory, antioxidant, immunostimulant, hepatoprotective, cytotoxic [7], anticancer [8], anti-allergic, antifungal [9], antiprotozoal [10], analgesic [11], and antinociceptive [12] activities. The mechanisms underlying these biological effects are still being uncovered and continue to be elucidated [13]. Importantly, reported adverse effects and toxicity remain minimal [14].

Most plant species have a modified chemical profile due to a multitude of environmental factors [15]. Propolis similarly varies in its composition, not only because it is a product derived from plants, but also due to changes in bees’ preference for certain plant sources and geographic regions [1,16,17]. The percentage concentration of apitherapeutic components also varies according to the bee species [18]. Nevertheless, propolis samples with distinct chemical profiles have been shown to elicit biological activities of similar type and magnitude [19].

Variations in composition are constrained by the foraging behavior of *Apis mellifera*, which selectively gathers resins from specific plant sources [20,21]. Consequently, the chemical profile of plants surrounding the hive directly determines the composition of propolis [22], resulting in differences in color, botanical origin, and chemical profile [23,24]. On this basis, categories of apiceuticals or bee products have been established according to different propolis types [23]. In Brazil, owing to the country’s vast size and biodiversity [24], there are thirteen defined groups, eight referring to brown propolis [25].

In Brazil, the types of brown propolis differ markedly in chemical composition [26], reflecting their diverse botanical origins [27]. Consequently, further research on Brazilian brown propolis is strongly recommended to ensure the development of safe and effective products for the national market [27].

Our research group has thoroughly studied this new type of propolis since 2019, demonstrating biological properties that make it a candidate for inclusion in the classification and typification system of Brazilian propolis, as well as potentially assisting Brazilian governmental organizations in establishing a new geographical indication for greenish-brown propolis from the mountainous regions of Alagoas in the northeast of Brazil. To our knowledge, no publications to date have addressed this variety, underscoring the importance of our investigation.

The aim of this study was to evaluate the seasonal variation of chemical markers, total flavonoid content, total phenolic content, flavanone and dihydroflavonol content, 2,2-diphenyl-1-picrylhydrazyl (DPPH•) radical scavenging capacity, and in vitro antimicrobial and antileishmanial activities of GBPUP, in addition to applying multivariate statistical analysis to identify the main markers responsible for the biological activities in GBPUP extracts.

## 2. Results

### 2.1. Identification of Chemical Markers by LC–ESI–Orbitrap–FTMS

The identification of compounds was accomplished using the base peak intensity chromatogram (*m*/*z* 148–2000) in MZmine 2, version 2.53, software, as shown in Table 1 and Supplementary Figure S1 (Section S2). Table 1 presents the 21 major constituents, which include flavanones and flavanonols (pinocembrin, pinostrobin, naringenin, pinobanksin), flavonols and glucosilated flavonols (quercetin, rutin, kaempferol, galangin), and the flavone chrysin. Hydroxycinnamic acid derivatives were also detected, notably caffeic acid, ferulic acid, *p*-coumaric acid, and artepillin C. Additional phenolic compounds identified comprised aromadendrin, catechin, and pinobanksin 3-acetate.

Preliminary characterization by LC–ESI–Orbitrap–FTMS in full-scan mode demonstrated that GBPUP propolis extracts are enriched in flavonoids (particularly flavanones and flavanonols) and hydroxycinnamic acid derivatives (artepillin C), with a significant representation of prenylated structures. Some compounds varied in retention time due to the difference between chromatographic systems (Shimadzu^®^, Tokyo, Japan) and (Thermo Fisher Sicentific^®^, Hemel Hempstead, UK) and a discrepancy in column C_18_ between laboratories. Subsequent quantitative analysis of 13 phenolic constituents was performed using UFLC–DAD–UV–Vis, enabling chromatographic peak confirmation via diode array detection. Quantification was conducted under external calibration conditions, employing standard calibration curves to ensure analytical robustness and reproducibility.

### 2.2. Quantification of Chemical Markers by UFLC–DAD–UV–Vis

**Figure 1:**
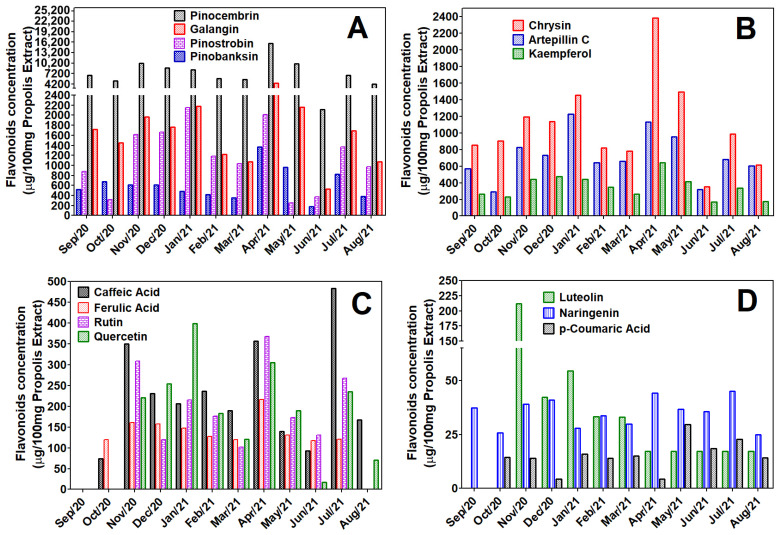
Determination of the flavonoid, flavanone, and hydroxycinnamic acid content of the greenish-brown propolis from União dos Palmares, Alagoas, Brazil using UFLC–DAD–UV–Vis. Quantification of chemical markers: (**A**) pinocembrin, galangin, pinostrobin, pinobanksin; (**B**) chrysin, artepillin C, and kaempferol; (**C**) caffeic acid, ferulic acid, rutin, and quercetin; (**D**) luteolin, naringenin, and *p*-coumaric acid. The concentrations were expressed as amount in micrograms of flavonoids or hydroxycinnamic acids present in 100 mg of GBPUP extract.

The concentration of GBPUP chemical markers during the 12 months, expressed in µg of each compound contained in 100 mg of GBPUP extract, is shown in Figure 1. The compounds analyzed belong to the category of hydroxycinnamic acids (artepillin C, caffeic acid, ferulic acid, and *p*-coumaric acid), flavonols and glucosilated flavonols (galangin, kaempferol, quercetin, and rutin), flavanones and flavanonols (pinocembrin, pinostrobin naringenin, and pinobanksin), and flavones (chrysin). GBPUP markers are different to those of Brazilian green propolis, which has caffeoylquinic acid, *p*-coumaric acid, hesperitin, kaempferide, drupanin, baccharin, kaempferol, and artepillin C among its major compounds [28]. Comparative studies of eight types of propolis between the southeastern region of Brazil (São Paulo, Minas Gerais, Paraná), the central-western region of Brazil (Goiás), and two states in northeastern Brazil (Piauí and Bahia) showed variations in the colors of the propolis analyzed. There are samples from different locations, some with deep green color, others dark, and others black; often, the green samples contain high levels of phenolic compounds (prenylated hydroxycinnamic acids like artepillin C, baccharin, drupanin, and caffeoylquinic acids), while the dark and black ones contain mostly triterpenoids (*β*-amyrin, *α*-amyrin, *β*-amyrinone, *α*-amyrinone, *β*-amyrin acetate, *α*-amyrin acetate, taraxerone, oleanene, pteron-14-en-7-one, Olean-14-en-3,28-dione) [29].

These preliminary findings demonstrate that the greenish-brown propolis from the Serras’ region of Alagoas, Brazil can possibly be considered an intraspecific variation of green propolis, dark propolis, or black propolis, which has not yet been studied, containing metabolites including flavanones (pinocembrin and pinostrobin), flavone (chrysin), flavanols (galangin and kaempferol), and prenylated hydroxycinnamic acids (artepillin C) as the major compounds.

### 2.3. Antimicrobial Activity

The broth microdilution assays generated MICs for all GBPUP extracts (Table 2), revealing consistent monthly activity from September 2020 to August 2021 for each tested microorganism. The antibacterial activity of Brazilian green propolis has been largely attributed to elevated levels of flavonoids and phenolic compounds, including galangin, kaempferol, pinostrobin, pinocembrin, quercetin, caffeic acid, and artepillin C, which have been associated with inhibition of bacterial RNA polymerase and disruption of cell membranes or cell walls [30,31,32]. In the present study, galangin and pinocembrin were detected at relatively high concentrations in the GBPUP extracts, suggesting that these flavonoids may contribute substantially to the observed antibacterial activity.

### 2.4. Antileishmanial Activity

**Figure 2:**
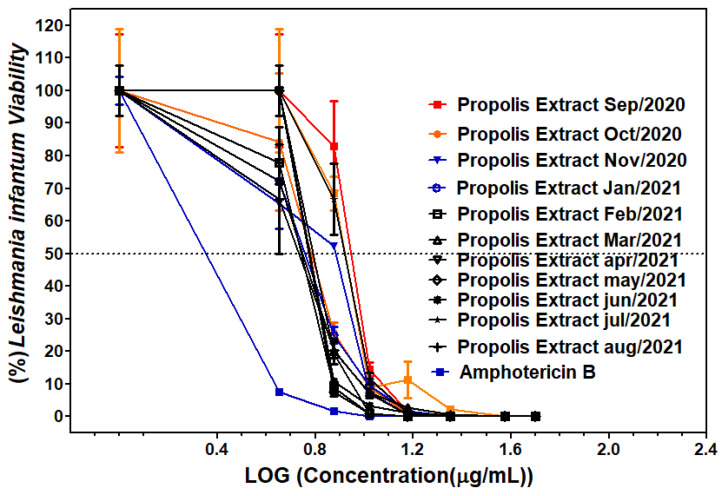
Determination of the viability of *Leishmania* (V.) *infantum* against greenish-brown propolis extract or amphotericin B using a normalized graph.

Greenish-brown propolis extracts from the União dos Palmares, Alagoas, Brazil, exhibited leishmanicidal activity in all samples tested and across the entire concentration range. Figure 2 shows the *Leishmania infantum* viability and IC_50_ values of the leishmanicidal assay for GBPUP extracts from Alagoas, Brazil. The IC_50_ values showed that the best IC_50_ values were between January and June, in the range of 5.38 to 6.74 µg/mL, as well as in August 2021 with an IC_50_ value of 5.35 µg/mL. In June, the value was 6.46 µg/mL, and between September and December, the IC_50_ values ranged from 6.15 to 8.78 µg/mL. Amphotericin B (positive control) showed an IC_50_ value of 1.96 µg/mL.

### 2.5. Multivariate Statistical Analysis

#### 2.5.1. DIABLO Model Using Circos Plot Analysis

**Figure 3:**
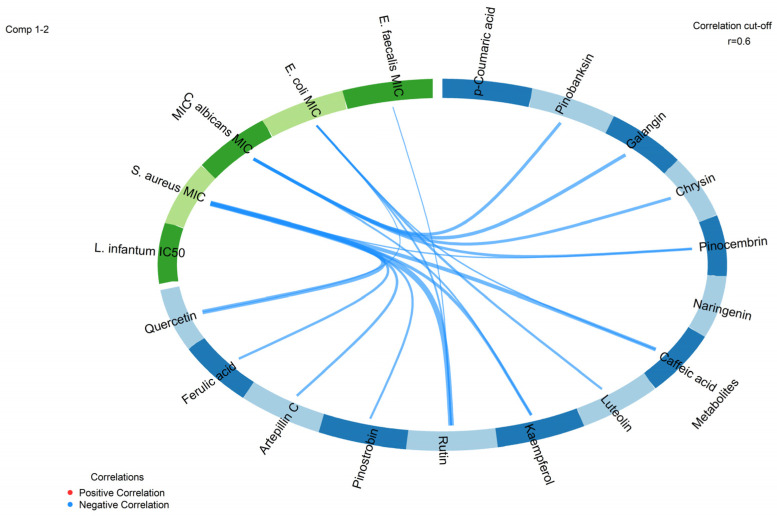
Circos plot analysis assessed the association between antibacterial (*S. aureus*, *E. faecalis*, *E. coli*) or antifungal activities (*C. albicans*) and the main phenolic or flavonoid metabolites of greenish-brown propolis using a cut-off of r > 0.60. Leishmanicidal activity was not observed in the Circos plot analysis within this cut-off range.

Circos plot analysis revealed a high association (r ≥ 0.7) between the chemical metabolite block and the MIC-based bioactivity block (Figure 3). Specifically, flavonoids and hydroxycinnamic acids were negatively correlated with the MIC bioactivity block for *Staphylococcus aureus*. A high negative correlation was observed for the metabolites rutin, quercetin, and caffeic acid, followed by artepillin C, kaempferol, and ferulic acid. Similarly, a high negative correlation was also observed between the metabolites galangin, pinobanksin, chrysin, and pinocembrin and the MIC values for *Candida albicans* in the bioactivity block. Moderate correlations were observed between the metabolite block and the bioactivity block when the cut-off point was reduced to r ≥ 0.65 and r ≥ 0.60. Specifically, luteolin, rutin, quercetin, and caffeic acid were negatively correlated with *Escherichia coli* MIC, while rutin was also negatively correlated with *Enterococcus faecalis* MIC. No associations above the cut-off were observed between the metabolite block and leishmanicidal activity. (Figure 3).

#### 2.5.2. ASCA Analysis

ASCA analysis revealed that geographical origin, i.e., apiary area, was the main structured source of metabolic variability, explaining 23.66% of the total variance (*p* < 0.001). In contrast, the antileishmanial activity (IC_50_) accounted for 5.67% of the total variance (*p* < 0.001), indicating a significant but comparatively smaller metabolic signature associated with bioactivity. The interaction between apiaries and IC_50_ was statistically significant but explained only 0.51% of the variance, suggesting that the relationship between metabolic composition and antileishmanial potency is largely consistent across geographical origins. Residual variation accounted for 69.51% of the total variance, reflecting intrinsic biological variability and potential minor compositional differences not explained by the experimental factors. Together, these findings demonstrate that although geographic origin is the primary determinant of global metabolic variability, a distinct subset of metabolites is associated with antileishmanial activity, supporting a bioactivity-linked metabolic signature in propolis.

**Figure 4:**
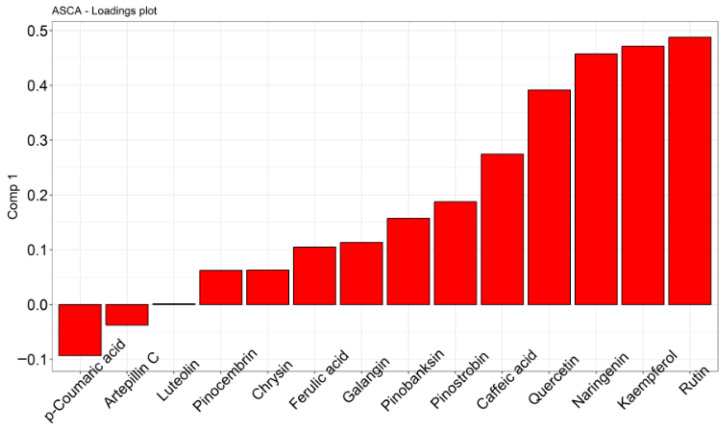
ASCA loading plot analysis shows the association between the metabolites of phenolic compounds and flavonoids quantified in greenish-brown propolis and leishmanicidal activity. Negative correlations demonstrate a higher probability of leishmanicidal activity, and zero correlations at +0.20 demonstrate intermediate leishmanicidal activity. Positive correlations (+0.3 to +1.0) are associated with a low probability of leishmanicidal activity.

The first ASCA component associated with the IC_50_ effect captured a structured metabolic gradient correlated with antileishmanial potency. Score plots revealed a continuous distribution of samples along the IC_50_ axis, supporting a linear association between metabolite composition and bioactivity. An ASCA scores plot with negative scores plotted in component 1 identified Mata dos Frios and Branquinha apiaries as primarily responsible for leishmanicidal activity, as shown in Figure 4. These apiaries present a phytochemical composition of metabolites that, as a whole, are promoting leishmanicidal activity.

The ASCA loadings plot analysis identified metabolites contributing to this component, indicating that specific chemical constituents are systematically associated with variations in IC_50_ values. Metabolites with negative loadings (−0.1) were associated with lower IC_50_ values (higher antileishmanial activity), suggesting their potential contribution to biological efficacy. In particular, p-coumaric acid and artepillin C were positioned in the negative loading region and showed an inverse relationship with both component scores and IC_50_ values, suggesting a potential contribution of these compounds to antileishmanial activity. Based on the loading plot between −0.0 and +0.2, we can establish a ranking of moderate bioactive compounds for leishmanicidal activity, which we can describe as luteolin with high association, pinocembrin, chrysin, ferulic acid, galangin, pinobanksin, and pinostrobin. In contrast, flavonoids such as rutin, kaempferol, naringenin, quercetin, and caffeic acid were associated with positive loadings (>0.30) and were therefore correlated with higher IC_50_ values, indicating a comparatively weaker association with antileishmanial potency in the analyzed samples (Figure 4).

## 3. Discussion

Several analytes were not detected at concentrations sufficient to permit inclusion in the seasonality study. For example, luteolin remained consistently low relative to other constituents and exhibited no temporal variation, except for a slight increase in November 2020. Likewise, catechin, 2-coumaric acid, resveratrol, coumarin, and liquiritigenin were observed only sporadically at trace levels (Figure 1). These findings suggest that the concentrations of these compounds were below the analytical method’s quantification limits, thereby precluding assessment of their seasonal fluctuations.

Among the 12 chromatograms of the crude extracts, the samples from January 2021 and April 2021 had the most peaks with a large area at λ 313–315 nm (Figure 1) and (hence the chromatogram selected for Supplementary Figure S5 in Section S2).

Temporal profiling revealed two distinct four-month cycles characterized by elevated marker abundance: October 2020–January 2021 and April–July 2021. Within these cycles, certain months deviated from the general pattern. Notably, June 2021 exhibited markedly reduced concentrations across multiple analytes, including galangin, kaempferol, pinocembrin, and chrysin, representing the lowest values observed in the dataset. Similarly, October 2020 displayed comparatively diminished levels within its respective cycle.

Interestingly, rutin, quercetin, ferulic acid, *p*-coumaric acid, and caffeic acid were at zero in September 2020, indicating that this was a month in which the GBPUP had relatively lower quality. Other months in which this occurred were October 2020, for rutin and quercetin, and August 2021, exclusively for rutin. Conversely, the months of January (first cycle) and April (second cycle) of 2021 had the highest quantities of all compounds, with only a few exceptions.

Among the quantified constituents, the flavanone pinocembrin was consistently the most abundant, reaching a maximum concentration of 15.80% in April 2021. Even at its lowest level (4.24% in August 2021), pinocembrin exceeded the content of all other markers across the study period. Given its prevalence, it is reasonable to hypothesize that GBPUP propolis may exert at least part of its biological activity through pinocembrin, which has been reported to possess anti-inflammatory, antimicrobial, antioxidant, anticancer, antifibrotic, and neuroprotective properties [33]. Pinostrobin, another major flavanone, shares many of these activities, but additionally demonstrates lipid-lowering, uric-acid-reducing, and wound-healing effects, with protective tropism for hepatic and gastrointestinal tissues [34]. The presence of these bioactive flavonoids underscores the pharmacological potential of GBPUP and warrants further investigation into its bioactivities, mechanisms of action, and pharmacokinetics.

Markers that, although in significantly lower quantities than pinocembrin, were found to be relatively abundant include artepillin C, pinobanksin, pinostrobin, galangin, and chrysin. The average artepillin C content was 4.31 ± 1.62 µg/mL or 7.19 ± 2.71 mg/g; the average *p*-coumaric acid content was 0.08 ± 0.04 µg/mL or 0.13 ± 0.07 mg/g; and the average pinocembrin content was 7449.00 ± 3379.93 µg/100 mg or 74.49 ± 32.47 mg/g.

The results of the DPPH• radical scavenging capacity assay were used to generate the graph in Supplementary Tabel S1 (Section S1), which shows the half-maximal inhibitory concentration (IC_50_) for the 12 months, observed in each month. Again, the seasonal variation showed two cycles of maximum antioxidant activity, indicating that this effect is a product of the previously quantified metabolites. The first cycle covered the period from October 2020 to January 2021, the second, from April to July 2021. Both comprised 4 months, totaling 8 months, during which GBPUP extracts demonstrated better bioactivity.

The average IC_50_ using the DPPH• method for the first cycle was 44.44 ± 3.13 µg/mL; the best, and also the second lowest, occurred in January 2021 at 39.66 µg/mL. This corroborates the results of other trials, in which January 2021 was the month of the first cycle with the highest concentrations. The second cycle had an average IC_50_ of 37.21 ± 4.58 µg/mL, and April 2021 was the month with the lowest IC_50_ (30.03 ± 4.08 µg/mL), results that are in accordance with the chemical assay of total flavonoids and, in part, with the content of total phenolic compounds and UFLC–DAD–UV results.

Melo et al. (2010) [35] proposed a classification for IC_50_ according to which propolis extracts have good antioxidant activity when their IC_50_ < 65 µg/mL, moderate when their IC_50_ < 152 µg/mL, and low when their IC_50_ > 152 µg/mL. Therefore, the average IC_50_ of GBPUP hydroethanolic extracts constitutes good antioxidant activity. There are, however, brief periods in which antioxidant activity becomes moderate, as in September 2020 and August 2021.

Overall, GBPUP extracts were more effective against Gram-positive bacteria than Gram-negative bacteria, which was also shown in the cut-off point of the Circos plot analysis (r > 0.70). MICs tended to improve during the second cycle (April–July 2021) compared to the first cycle (October 2020–January 2021), although high MICs from October–December 2020, particularly against *E. coli* and *C. albicans*, precluded precise cycle-based comparisons. January 2021 corresponded to the lowest MICs across all microorganisms (Table 2), coinciding with the highest total flavonoid content (Table S1 in Section S1). Interestingly, the Circos plot analysis showed strong activity of the metabolites rutin, quercetin, and caffeic acid with a cut-off point of r > 0.70, followed by artepillin C, kaempferol, and ferulic acid with a cut-off of 0.70 > r > 0.60, in inhibiting the Gram-positive bacterium *S. aureus*.

*Enterococcus faecalis* was consistently susceptible to GBPUP, with growth inhibited at 128 µg/mL and peaking at 64 µg/mL in January 2021, aligning with maximal flavonoid levels (Table 2 and Table S1 in Section S1). These MICs were lower than those reported for ethanolic extracts of brown propolis from various Brazilian regions (1000 µg/mL–860 µg/mL), and comparable to hydroethanolic extracts showing bacteriostatic activity in 36% of samples at 156–625 µg/mL [36,37,38,39]. While phenolic compounds are known to contribute to the antimicrobial activity of propolis and may be influenced by extraction efficiency, temporal variation detected in quantitative chemical analyses was not observed here by pronounced shifts in MIC values against *E. faecalis*. Circos plot analysis demonstrated that the flavonoid rutin (quercetin-3-rutenoside) showed a moderate association with antibacterial activity against *E. faecalis* (cut-off point r > 0.60). Some scientific studies demonstrate the antibacterial and antibiofilm activity of rutin and kaempferol against *E. facalis*, with some applications of this flavonoid to reduce clinical strains of *E. facalis* in the dental field and to reduce urinary tract infections associated with *E. faecalis* [40,41,42,43].

*Escherichia coli* was the least susceptible microorganism to the GBPUP extracts, consistently displaying the highest MIC values among those tested. The lowest MIC (256 µg/mL) was observed in January 2021, whereas an MIC of 512 µg/mL predominated across most of the sampling period, being detected in nine months, including February through August 2021. In contrast, reduced bacteriostatic activity was recorded in October and December 2020, when growth inhibition required 1024 µg/mL. These data suggest relatively limited temporal variation in GBPUP activity against *E. coli* over the study period (Table 2).

The MICs obtained for *Candida albicans* varied across the sampling period. The lowest MICs (64 µg/mL) were observed in January and April 2021, indicating higher antifungal activity in these months. For the remaining period, MICs ranged from 128 to 256 µg/mL, with the lowest values (128 µg/mL) recorded from April to August 2021. Higher MICs (256 µg/mL) were observed in September and November 2020, as well as in February and March 2021 (Table 2). Circos plot analysis also showed a high correlation (r > 0.7) between the metabolites galangin, pinobanksin, chrysin, and pinocembrin and the MIC bioactivity against *Candida albicans*. These temporal patterns broadly coincided with variations in the chemical profiles of the extracts, including flavonoid composition, total phenolic and flavonoid contents, and antioxidant capacity as assessed by DPPH• scavenging assays (Supplementary Table S1). In comparison with previous reports on Brazilian brown propolis, GBPUP extracts displayed measurable antifungal activity against *C. albicans*, whereas several studies have reported no inhibitory effects for other preparations.

Studies of the leishmanicidal activity of Brazilian red propolis have shown IC_50_ values between 25 µg/mL and 38 µg/mL [44,45]. The leishmanicidal activity of Bulgarian propolis extract was demonstrated against the *L. amazonensis*, *L. chagasi*, and *L. major* species, with an IC_50_ value ranging from 2.8 to 41.3 μg/mL. The excellent leishmanicidal activity of Bulgarian propolis can be attributed to the various flavonoids present in its extract, including pinobanksin esters, pinocembrin, chrysin, and some phenolic acids and esters [46]. Scientific research by Da Silva et al. (2013) [47] showed that Brazilian green propolis displays leishmanicidal activity at concentrations ranging from 5 to 100 μg/mL in in vitro cytotoxicity studies at 24, 96, and 168 h.

Considering the significant antileishmanial activity of GBPUP, the bioactive compounds identified by UFLC–DAD–UV, and the literature, several mechanisms of action can be proposed. The flavonol quercetin, for example, inhibits the enzyme arginase of *Leishmania* (L.) *amazonensis*, which is required for parasite proliferation and detoxification of reactive oxygen species via trypanothione. The absence of sugar moieties in its structure allows interactions between the catechol and ketone groups of the C ring with L-arginine (substrate) and Mn^2+^ (cofactor). In addition, it activates topoisomerase II, causing cleavage of the mitochondrial kDNA, and inhibits the parasite ribonucleotide reductase, factors that lead to apoptosis [48].

In another investigation, the flavonol kaempferol 3,7-di-O-methyl ether exhibited an IC_50_ of 10.5 µM against the amastigote forms of *Leishmania* (L.) *amazonensis*, leading to G0/G1 cell cycle arrest [49]. Additionally, rutin (quercetin-3-rutinoside) showed selective inhibition of the enzyme Ld3MST, compromising the supply of sulphide and, consequently, cysteine to *L. donovani*. Thus, a collapse of the parasite’s antioxidant defenses is likely [50].

Some flavones have been proposed as having leishmanicidal activities, such as luteolin and apigenin, whose structures are very similar to that of the flavone chrysin, quantified in this study. In silico studies suggest that luteolin also acts on topoisomerase II, but via inhibition, whereas molecular docking results indicate high-affinity binding to lanosterol 14α-demethylase (CYP51), thereby interfering with ergosterol synthesis in *L. infantum*. In in vivo and in vitro studies, apigenin, similarly to quercetin, inhibited arginase in *L. amazonensis* and *L. donovani*, blocking the synthesis of polyamines essential for parasite survival [51].

Both artepillin C and *p*-coumaric acid, hydroxycinnamic acids, showed activity against promastigotes of *Leishmania* (L.) *amazonensis* at concentrations of 3.12 and 6.25 µM. On the other hand, *p*-coumaric acid exhibited promising activity against amastigotes, with an IC_50_ of 3.12 µM [52]. Caffeic acid, detected in GBPUP, induces apoptosis through a caspase-independent pathway, causing DNA fragmentation, in addition to exerting immunomodulatory effects that stimulate the intracellular killing of amastigotes [53]. In a comparative study involving a hydroalcoholic extract of geopropolis and different fractions, phenolic acids showed high efficacy against promastigote forms, especially gallic acid, which inhibited them with an IC_50_ of 14.48 µg/mL [54], indicating the possible role of this class of compounds in the effectiveness of GBPUP. Luteolin itself binds to CYP51 with greater affinity than fluconazole. It gains stability through hydrogen bonds with the residues Tyr115, Ala286, Met357, and Met459; van der Waals interactions with Tyr102, Phe109, Phe289, Val356, Leu358, and Val460; and binding to the HEME group [53].

Luteolin exhibited 83% in vitro inhibition of *Leishmania amazonensis* arginase and one of the lowest IC_50_s of around 9 µM compared to other isolated compounds used in the study, such as galangin, which had an IC_50_ of 100 µM. Molecular docking showed that these compounds interact with residues in the enzyme’s catalytic site, where Mn^2+^ is located. Furthermore, no toxicity was observed in mammalian arginase (ARG-1), indicating the compounds’ good selectivity and favorable safety profile for GBPUP [55]. Galangin proved relevant in a rigorous investigation of the mechanisms of leishmanicidal activity. In the research, the authors not only obtained the best IC_50_ values in vitro, 53.09 µM in promastigotes and 20.59 µM in intracellular amastigotes of *L. amazonensis*, but also identified by transmission electron microscopy that galangin causes intense damage to the ultrastructure of the parasite, with the presence of mitochondrial swelling, cytoplasmic lipid bodies, and plasma membrane blebs (surface blebbing) [56].

An example of this is 2′-hydroxyflavanone, which has demonstrated efficacy against *L. amazonensis* in vivo and in vitro. Not only was the wild-type parasite inhibited in the study, with IC_50_ values of 20.96 μM and 3.09 μM against the promastigote and intracellular amastigote forms, respectively, but the antimony-resistant parasite was also inhibited in a dose-dependent manner, with IC_50_ values of 24.34 μM and 3.36 μM against the promastigote and intracellular amastigote forms, respectively. Furthermore, in BALB/c mouse assays using wild-type or antimony-resistant *L. amazonensis* promastigotes, 2′-hydroxyflavanone decreased lesion size and parasite load by 98.8% or 99% [57].

Pinostrobin, pinocembrin, tectocrisin, and galangin 3-methyl ether were isolated from the *Lychnophora markgravii* extract and were found to decrease the viability of *L. amazonensis* amastigotes, with respective IC_50_ values of 0.31, 3.45, 0.56, and 2.89 µM. Finally, the authors suggest that the arrangement of hydroxyl groups causes the differences in activity between the compounds. For instance, pinostrobin and galangin 3-methyl ether significantly reduced parasite viability at a concentration of 0.5 mg/mL. The authors therefore explain that the arrangement of hydroxyl groups in the flavonoid structure is fundamental to the observed difference in activity [58].

Another study presented IC_50_ values for various compounds isolated from propolis extracts, including pinobanksin 3-O-acetate, pinostrobin, galangin, chrysin, and pinocembrin, which were tested against wild-type *L. mexicana* and *L. mexicana* C12Rx (resistant to miltefosine). Thus, a propolis sample with a chemical profile similar to GBPUP was found to contain compounds with antileishmanial activity that act through different mechanisms to standard drugs, as they were active even against resistant parasites [59].

In vitro studies showed that the flavanone pinostrobin had an IC_50_ of 13.61 μg/mL (50.39 μM) against *L. braziliensis*. In hamster models of cutaneous leishmaniasis, it achieved a 50% cure rate and reduced ulcer size by 84–87%. This constituted a significant improvement in 40% of the animals [60]. In a leishmanicidal assay with *Leishmania tropica* promastigotes, ferulic acid demonstrated dose-dependent inhibitory activity ranging from 0 to 60.4%, resulting in an IC_50_ of 11.1 µg/mL. Molecular docking studies revealed that ferulic acid forms hydrogen bonds with residues SER-234, GLU-166, THR-129, and THR-130, as well as van der Waals interactions with TRP-61, ALA-128, and PHE-177, within leishmanolysin GP63 [61].

## 4. Materials and Methods

### 4.1. Chemicals

The analytical standards for ferulic acid, caffeic acid, *p*-coumaric acid, 2-coumaric acid, resveratrol, catechin, quercetin, rutin, galangin, luteolin, liquiritigenin, pinobanksin, pinocembrin, pinostrobin, naringenin, kaempferol, and chrysin were purchased from Sigma-Aldrich (St. Louis, MO, USA), and artepillin C was purchased from Chemfaces (Wuhan, Hubei, China). Both HPLC-grade methanol and formic acid were purchased from J. T. Baker (Mallinckrodt Baker Inc, Mexico city, Mexico), while Milli-Q and Reverse Osmose water were produced at the Pharmaceutical and Food Analysis Laboratory of the Federal University of Alagoas, in Maceió/AL.

### 4.2. Propolis from União Dos Palmares Region, Alagoas, Northeast Brazil

This research was previously registered at the National System for the Management of Genetic Heritage and Associated Traditional Knowledge (SISGEN/CGEN/Brazilian Ministry of the Environment) for access, collection, and/or traditional knowledge (TK) under codes A2F5549 and AA7727B. GBPUP samples were donated by the company “Zumbi dos Palmares” from five different apiaries (Mata dos Frios, geographical coordinates of south latitude: 9°10.300′, west latitude: 36°02.060′; Serra da Barriga, geographical coordinates of south latitude: 9°10.250′, west latitude: 36°05.200′; Mata Microondas, geographical coordinates of south latitude: 9°13.324′, west latitude: 36°01.550′; Sueca, geographical coordinates of south latitude: 9°02.560′, west latitude: 35°56.541′; and Branquinha, geographical coordinates of south latitude: 9°13.324′, west latitude: 35°59.110′) in the Serras’ region of the municipality of União dos Palmares in Alagoas, Brazil. Monthly, the Zumbi dos Palmares company chose one of the apiaries (Mata dos Frios, Serra da Barriga, Mata Microondas, Sueca, and Branquinha) to collect only one propolis sample from the highest-producing hive. Then, the samples, ranging from 38 g to 91 g, always taken from the same hive, were labeled with apiary name and date, stored in an inert container, transported, and subjected to seasonal analysis of chemical and biological characterizations at the Federal University of Alagoas.

The propolis raw material was removed from the same hive every 28 to 30 days during the procedure of inspecting the hives. There was no variability related to propolis aging during the seasonal study. The same beekeeper was responsible for sampling, collecting, and transporting the propolis samples.

This monthly procedure resulted in 12 samples, covering the Zumbi dos Palmares company’s apiaries and encompassing the period from September 2020 to August 2021. The GBPUP samples were sanitized and stored in a refrigerator (4 to 8 °C) under light protection for a maximum of 30 days, before the maceration extraction process began. Supplementary Table S1 provides detailed information on sample origin throughout this seasonal cycle.

### 4.3. Sample Preparation

Samples were extracted using the maceration method in two 48 h cycles. For each seasonal sample, 15 g of crude propolis was soaked in 50 mL of 92.8% (*w*/*v*) hydroethanolic solution, with vigorous shaking applied upon initial contact between the solvent and the sample. The resulting supernatants were stored at 4 °C under refrigeration. After 7 days, the waxes precipitated and were removed by simple filtration. Solvents were removed by rotary evaporation, yielding crude GBPUP extracts, which were subsequently stored at −10 °C in a freezer. An amount of 10 g (66% yield) of extract was obtained during the maceration extraction process of GBPUP propolis extracts.

### 4.4. Qualitative Analysis of the GBPUP Extracts Using LC–ESI–Orbitrap –FTMS

The analytical standards (ferulic acid, caffeic acid, *p*-coumaric acid, 2-coumaric acid, artepillin C, resveratrol, catechin, quercetin, rutin, galangin, luteolin, liquiritigenin, pinobanksin, pinocembrin, pinostrobin, naringenin, kaempferol) were weighed ≅ 1.00 mg in analytical balance.

The LC/MS analysis in single-quadrupole mode was performed by the company Apex Science located in the city of Campinas-SP, Brazil. Analysis was carried out using LTQ Orbitrap™ equipment (Thermo Fisher Scientific, Hemel Hempstead, UK), set at a resolution of 30,000, +/− mode, with a mass range 160–2000 *m*/*z*.

The mobile phase consisted of 0.1% formic acid (A) in and methanol (B), (v:v) in gradient mode, and the stationary phase was a C18 column (150 × 4.6 mm, 5 µm) at a flow rate of 300 µL/min in gradient mode similar to the quantification mode using UFLC–DAD–UV–Vis.

Mass spectrometry (MS) data were processed using MZmine 2, version 2.53, software (http://mzmine.sourceforge.net/), URL (accessed on 20 April 2026). Peaks with an intensity of 1 × 10^4^ or higher were considered. For empirical formula determination, carbon (C), hydrogen (H), and oxygen (O) were included, with limits of up to 100 carbons, 200 hydrogens, and 50 oxygens per formula. The formula prediction function was applied only to ions with predicted molecular weights. A standardized laboratory procedure was used to remove adducts, complexes, and artifacts. The precise masses of sample components were then compared against online databases, including PubChem and ChemSpider. Analytical standards were used to confirm intensities and exact masses of the main peaks, which were established for a chromatography profile of GBPUP chemical markers. A table with the main peaks obtained in base peak intensity and exact mass was created. The chromatographic profile of LC–ESI–Orbitrap–FTMS using function base intensity peak versus retention time was acquired using MZmine software (Supplementary Figure S1).

### 4.5. Quantification of Chemical Markers by Chromatography

Chemical markers of GBPUP extracts were determined using ultra-performance liquid chromatography coupled to a diode array detector (UPLC–DAD). The device used for this purpose, a (Shimadzu, Tokyo, Japan), consisted of a Nexera model LC-20ADXR high-pressure pump, a DGU-20A3R degasser, a SIL-20AXR autoinjector, a CTO-20A column, SPD-M20A diode array detectors, a controller CBM-20A, and LabSolutions LC software, CL version.

The mobile phase consisted of 0.1% formic acid (solvent A) and methanol (solvent B), pumped at a flow rate of 0.3 mL/min. The stationary phase was a reversed phase (C18, 150 × 4.6 mm; 5 µm) on a Kinetex column (Phenomenex^®^, Torrance, CA, USA). The oven was maintained at 33 °C throughout all analyses. Elution was carried out using the following solvent gradient: 70% A (0–5 min), 64% A (5–8 min), 58% A (8–11 min), 52% A (11 –14 min), 52% B (14–20 min), 56% B (20–24 min), 62% B (24–28 min), 68% B (28–32 min), 72% B (32–36 min), 90% B (36–40 min), and 100% B (40–44 min), then returning to the initial gradient of 70% A (44–47 min). The injection volume was programmed to 2 µL.

In order to prevent some of the analytical standards from co-eluting during the preparation of their calibration curve, they were divided into two mixtures, which covered concentrations of 0.15 µg/mL, 0.3 µg/mL, 0.5 µg/mL, 2 µg/mL, 5 µg/mL, 15 µg/mL, 30 µg/mL, 60 µg/mL, and 75 µg/mL. It was necessary to dilute each standard to 1 mg/mL by weighing 1 mg of solute and dissolving it in 10 mL of HPLC-grade methanol. The first mixture of analytical standards was composed of catechin, caffeic acid, ferulic acid, 2-coumaric acid, naringenin, pinobanksin, kaempferol, pinocembrin, galangin, and pinostrobin; the second mixture of *p*-coumaric acid, rutin, resveratrol, coumarin, liquiritigenin, quercetin, luteolin, and chrysin. The analytical standard artepillin C was quantified separately. The linearity was demonstrated using calibration curve y = A ∗ x + B, and R^2^ ≥ 0.9800, where A is slope and B is y intercept. Both analytes gave a linear response as follows: caffeic acid (Y = 18,633X − 6042.4; R^2^ = 0.9996), ferulic acid (y = 18,893x − 10,592; R^2^ = 0.9993), *p*-coumaric acid (y = 23,534x − 620.25; R^2^ = 0.9999), artepillin C (y = 29,438x − 19,172; R^2^ = 0.9986), naringenin (y = 11,739x − 654.06; R^2^ = 0.9992), pinobanksin (y = 17,442x + 2028.5; R^2^ = 0.9989), kaempferol (y = 13,907x − 8664.4; R^2^ = 0.9973), pinocembrin (y = 10,927x − 930.34; R^2^ = 0.9993), galangin (y = 12,660x − 4097; R^2^ = 0.9997), pinostrobin (y = 12,377x − 7392.6; R^2^ = 0.9979), rutin (y = 3812.4x + 313.47; R^2^ = 0.9798), quercetin (y = 8403.2x − 864.88; R^2^ = 0.9984), luteolin (y = 10,002x − 891.3; R^2^ = 0.9948), chrysin (y = 23,349x − 12,730; R^2^ = 0.9989). The compounds had their peak area measured at the wavelength at which they showed maximum wavelength absorption (λmax): 277, 291, or 315 nm (Supplementary Figures S2–S4).

Methanolic extracts of GBPUP at 5 mg/mL were prepared by dissolving 50 mg of GBPUP extract in 10 mL of HPLC-grade methanol. Then, they were diluted to 600 µg/mL and filtered using 0.22 µm cellulose acetate membrane so that they could be injected into the chromatograph. Retention time (RT), diode array UV–Vis spectrum profile, and λmax (nm) were compared to those of standards, confirming the identity of the compounds (Supplementary Figure S5). The area (Y = intercept) of each identified metabolite (flavonoid or hydroxycinnamic acid) was correlated with its respective concentration in µg/mL determined by (X = slope of the calibration curve). Then, the concentration in µg/mL was converted into percentage values (%) of the metabolite concentration in the GBPUP extract by dividing by the nominal concentration (600 µg/mL) and multiplying by 100, also expressed as µg of metabolite/0.1 mg of GBPUP extract. The percentage values were multiplied by 1000 to obtain a concentration of 1000 µg/100 mg of GBPUP extract. Results were reported in µg of metabolites (flavonoid or hydroxycinnamic acid) quantified in UFLC–DAD–UV–Vis/100 mg GBPUP extract.

### 4.6. Antimicrobial Activity

The antimicrobial activity of the 12 GBPUP extracts was evaluated by broth microdilution to determine minimum inhibitory concentrations (MICs) against *Staphylococcus aureus* (ATCC 25923), *Enterococcus faecalis* (ATCC 29212), *Escherichia coli* (ATCC 25922), and *Candida albicans* (ATCC 90028), following CLSI guidelines (M07 for bacteria, M27 for yeast). Bacterial strains were recovered from lyophilized cultures in brain–heart infusion (BHI) broth, incubated at 35 ± 2 °C for 18–24 h, and standardized to 0.5 McFarland (1–2 × 10^8^ CFU/mL) in sterile saline, then diluted 1:10 to achieve 5 × 10^5^ CFU/mL per well. *C. albicans* colonies were cultured on Sabouraud dextrose agar at 35 ± 2 °C for 18–48 h, transferred to 3 mL RPMI-1640 broth, vortexed, and adjusted to 0.5 McFarland (1–5 × 10^6^ CFU/mL), then diluted 1:50 to obtain 2 × 10^4^ CFU/mL per well [62].

GBPUP extracts were reconstituted in 2% DMSO at 2000 µg/mL. Serial twofold dilutions were performed in sterile 96-well U-bottom microplates, yielding final concentrations of 1.000–7.81 µg/mL. Then, wells received 5 µL of bacterial or 50 µL of yeast inoculum, which were prepared in Mueller–Hinton broth (MHB) and RPMI-1640 broth, respectively [63]. Each plate included negative controls (broth with 2% DMSO and inoculum), viability controls (broth with inoculum), sterility controls (broth only), and positive controls (ciprofloxacin at concentration of 0.125 µg/mL for *S. aureus*, 0.25 µg/mL for *E. faecalis*, 0.0625 µg/mL for *E. coli*; fluconazole at concentration of 0.5 µg/mL for *C. albicans*). Plates were incubated at 35 ± 2 °C for 24 h. Microbial growth was assessed by adding 20 µL of 0.5% MTT solution to each well, followed by incubation at 35 °C for 3 h before measuring growth.

### 4.7. Antileishmanial Activity

Promastigote forms of *Leishmania* (L.) *infantum* BR2000 stains donated by Fiocruz Pernambuco (Recife, Brazil) were obtained from a certified reference collection and maintained under standardized axenic conditions. Parasites were cultured in a biphasic Schneider’s/Novy–MacNeal–Nicolle (NNN) medium system, supplemented with 10% heat-inactivated fetal bovine serum (FBS), 50 U/mL penicillin, and 50 µg/mL streptomycin to prevent bacterial contamination. Cultures were incubated at 26 °C in a biochemical oxygen demand (BOD) incubator, conditions that mimic the physiological environment of the insect vector and are widely adopted in antileishmanial drug screening protocols.

GBPUP extracts were accurately weighed and subjected to a controlled solubilization protocol to ensure physicochemical homogeneity and biological reproducibility. GBPUP extracts were dispersed in Tween 20 (100 µL) and sonicated for 20 min in an ultrasonic bath. Subsequently, ultrapure reverse-osmosis water (900 µL) was added (10:90, *v*/*v*) to obtain a primary stock solution at 10,000 µg/mL. Serial dilutions were prepared in Schneider’s medium supplemented with 10% FBS to produce working concentrations ranging from 50 to 4.5 µg/mL (final volume: 1 mL per concentration). Importantly, the final concentration of Tween 20 did not exceed 0.5% (*v*/*v*), a threshold previously validated as non-toxic to Leishmania promastigotes and therefore not interfering with parasite viability or growth kinetics.

Antileishmanial activity was assessed following a validated microplate-based protocol adapted from established methodologies reported by Rocha et al. (2009) [64] and Do Nascimento et al. (2016) [45]. Log-phase procyclic promastigotes were adjusted to a density of 4 × 10^6^ cells/mL and dispensed (100 µL) into 96-well microplates containing Schneider’s medium supplemented with 10% FBS. Subsequently, 100 µL of each test concentration of the GBPUP extracts was added, yielding final concentrations of 50, 37.5, 22.5, 15, 10, 7.5, and 4.5 µg/mL. Amphotericin B was employed as a positive reference drug across the same concentration range, serving both as an internal quality control and a benchmark for comparative potency. Negative controls consisted of parasite cultures exposed to the solvent system alone, under identical experimental conditions. Plates were incubated at 26 °C in a BOD incubator for 24 h, a time frame sufficient to detect early cytostatic and cytocidal effects on promastigote proliferation without inducing nutrient depletion artifacts.

Following incubation, parasite morphology and general viability were initially assessed qualitatively using an (inverted microscope,, Nikon, Eclipse NI-U, Tokyo, Japan) allowing rapid identification of structural alterations, motility impairment, and cell rounding—hallmarks of leishmanicidal stress. Quantitative evaluation was subsequently performed by direct parasite counting using a (Neubauer hemocytometer, Kasvi, Pinhais, Paraná, Brazil) under an optical microscope at 40× magnification. Growth inhibition was expressed as the percentage reduction in parasite density relative to untreated controls, and dose–response curves were constructed to calculate the inhibitory concentration required to reduce parasite growth by 50% (IC_50_). Results were reported as mean IC_50_ values, ensuring statistical robustness and facilitating inter-study comparability.

### 4.8. Statistical Analysis

Results were expressed as mean ± standard deviation. The statistical calculations of leishmania viability assay were generated using the non-linear package of GraphPad Prism 9^®^ software. The statistical assessment of total phenol content, total flavonoid content, total flavanone content, and antioxidant activity was conducted using a one-way ANOVA test, specifically the Bonferroni means multiple-comparison test, which showed significant difference at a *p*-value < 0.05.

To investigate relationships between variables, multivariate analysis was conducted using 12 samples distributed across the five apiaries. The sample composition included seven samples from the Mata dos Frios apiary, two samples from Branquinha, and one sample each from the Mata Microondas, Serra da Barriga, and Sueca apiaries. Detailed descriptions of the multivariate analyses applied are provided below.

#### 4.8.1. Data Integration Analysis for Biomarker Discovery Using Latent Components

To investigate the coordinated relationships between metabolite composition and antimicrobial activity, a multiblock integration analysis was performed using the DIABLO (data integration analysis for biomarker discovery using latent components) framework implemented in the mixOmics R package (R version 4.3.3) [65]. Two data blocks were included in the analysis: (i) a metabolite block comprising quantified chemical variables and (ii) a bioactivity block containing minimum inhibitory concentration (MIC) values for *Staphylococcus aureus*, *Enterococcus faecalis*, *Escherichia coli*, and *Candida albicans*, together with IC_50_ values for *Leishmania infantum*. All variables were centered and scaled to unit variance prior to model fitting to ensure comparability across datasets. Associations between selected variables from different blocks were evaluated based on pairwise correlations, and highly correlated features (|r| ≥ 0.7) were visualized using a Circos plot. For comparative purposes, additional correlation matrices were constructed using correlation coefficient thresholds of r = 0.65 and r = 0.60, enabling visualization of moderate-to-strong associations between metabolites and bioactivity parameters.

#### 4.8.2. ASCA Analysis

Multivariate analysis was performed using ANOVA simultaneous component analysis (ASCA) implemented in the HDANOVA R package [66,67]. Prior to analysis, metabolite variables were autoscaled (mean-centered and unit-variance-scaled). A factorial model including apiaries, *Leishmania infantum* IC_50_, and their interaction was fitted: Metabolites*Apiaries* *Leishmania infantum* IC_50_. Type II sums of squares with sum coding were used under a restricted least-squares framework. Statistical significance of each effect was assessed using 1000 permutation tests. ASCA scores and loadings were extracted to evaluate the metabolic patterns associated with each factor.

## 5. Conclusions

In summary, pinocembrin was the major bioactive compound in GBPUP. Although artepillin C, pinobanksin, pinostrobin, galangin, and chrysin were present at lower concentrations than pinocembrin, their levels were consistently significant throughout the 12 months. High concentrations of total phenolic compounds, total flavonoids, flavanones, and dihydroflavonols were observed, along with strong DPPH• radical scavenging capacity, good antileishmanial activity, and greater sensitivity of Gram-positive bacteria to the extract. In addition, January 2021 was notable for a peak in total flavonoid content, which corroborated the leishmanicidal activity and the MIC values observed in that month. Circos plot analysis showed that specific metabolites were responsible for the high activity against *S. aureus* (artepillin C, kaempferol, and ferulic acid) and *C. albicans* (galangin, pinobanksin, chrysin, and pinocembrin) and moderate antibacterial activity against *E. faecalis* (rutin) and *E. coli* (luteolin, rutin, quercetin, and caffeic acid). ASCA analysis showed a strong correlation between the metabolites (*p*-coumaric acid, artepillin C, luteolin) and leishmanicidal activity.

This first investigation of seasonality not only reveals the period in which the bioactive potential of GBPUP extracts can be best exploited but also indicates a propolis variety with distinct chemical and biological properties. Therefore, these findings provide a relevant scientific basis for future studies and support their applicability in the development of bioactive or biomedical products and materials.

## Figures and Tables

**Table 1:** Confirmation of the chemical markers in samples of greenish-brown propolis extract (GBPUP) from União dos Palmares, Alagoas, Brazil during a seasonality study using LC–ESI–ORBITRAP–FTMS data and MZmine software.

Peak	R.T. (min.)	[M-H]^−^ or [M-H]^+^ (*m*/*z*)	MW	Formula	Area	Compound
1	1.28	169.0916	170.0215	C_7_H_6_O_5_	5.40 × 10^4^	Gallic acid
2	2.18	289.0711	290.0790	C_15_H_14_O_6_	2.50 × 10^4^	Catechin
3	2.38	179.0351	180.0422	C_9_H_8_O_4_	8.60 × 10^4^	Caffeic acid
4	3.50	193.0502	194.0579	C_10_H_10_O_4_	8.10 × 10^4^	Ferulic acid
5	3.97	165.0554	164.0473	C_9_H_8_O_3_	5.40 × 10^4^	2-coumaric acid
6	5.75	163.0243	164.0473	C_9_H_8_O	3.90 × 10^4^	*p*-coumaric acid
7	6.60	229.0675	228.0786	C_14_H_12_O_3_	2.20 × 10^4^	Resveratrol
8	9.48	609.0450	610.5000	C_27_H_30_O_16_	3.90 × 10^6^	Rutin
9	9.51	257.0805	256.0735	C_15_H_12_O_4_	2.60 × 10^5^	Liquiritigenin
10	10.30	303.0503	302.0426	C_15_H_10_O_7_	8.40 × 10^4^	Quercetin
11	10.70	273.0624	272.0684	C_15_H_12_O_5_	1.10 × 10^5^	Naringenin
12	13.68	272.0650	272.0684	C_15_H_12_O_5_	1.00 × 10^7^	Pinobanksin
13	13.70	285.0296	286.0477	C_15_H_10_O_6_	1.30 × 10^4^	Luteolin
14	14.37	285.0296	286.0477	C_15_H_10_O_6_	3.20 × 10^5^	Kaempferol
15	16.30	287.0575	288.0633	C_15_H_12_O_6_	9.3 × 10^5^	Aromadendrin
16	17.86	255.0809	256.0735	C_15_H_12_O_4_	1.90 × 10^8^	Pinocembrin
17	19.10	313.0731	314.0790	C_17_H_14_O_6_	3.1 × 10^5^	Pinobanksin-3-O-acetate
18	20.30	255.0517	254.0579	C_15_H_10_O_4_	2.20 × 10^5^	Chrysin
19	21.90	269.0464	270.0528	C_15_H_14_O_5_	6.2 × 10^7^	Galangin
20	25.97	271.099	270.2800	C_16_H_14_O_4_	1.2 × 10^8^	Pinostrobin
21	30.40	301.142	300.1725	C_19_H_24_O_3_	9.3 × 10^7^	Artepillin C

R.T.: retention time (min), MW: molecular weight.

**Table 2:** Minimum inhibitory concentrations (MICs) of the greenish-brown propolis extract (GBPUP) in the broth microdilution assay.

Microorganisms	Minimum Inhibitory Concentration (µg/mL)											
	Seasonal Propolis Samples											
	Sep/20	Oct/20	Nov/20	Dec/20	Jan/21	Feb/21	Mar/21	Apr/21	May/21	Jun/21	Jul/21	Aug/21
*Staphylococcus aureus* (ATCC 25923)	256	256	128	256	64	256	128	128	128	128	128	256
*Enterococcus faecalis* (ATCC 29212)	128	128	128	128	64	128	128	128	128	128	128	128
*Escherichia coli* (ATCC 25922)	512	1024	512	1024	256	512	512	512	512	512	512	512
*Candida albicans* (ATCC 90028)	256	128	256	128	64	256	256	64	128	128	128	128

Positive controls (ciprofloxacin at concentration of 0.125 µg/mL for *S. aureus*, 0.25 µg/mL for *E. faecalis*, 0.0625 µg/mL for *E. coli*; fluconazole at concentration of 0.5 µg/mL for *C. albicans*).

## Data Availability

The original contributions presented in this study are included in the article/Supplementary Material. Further inquiries can be directed to the corresponding author.

## Supplementary Materials

The following supporting information can be downloaded at: https://www.mdpi.com/article/10.3390/molecules31091447/s1, Supplementary Figure S1. Superposition of the chromatograms of the GBPUP seasonal extract samples for the months January 2021, February 2021, March 2021, and April 2021; Supplementary Figure S2. The chromatogram of mixture 1 of the analytical standard at a concentration of 75 µg/mL was detected using UFLC–DAD–UV–Vis with a specific wavelength of 291 nm for flavanones; Supplementary Figure S3. The chromatogram of mixture 2 of the analytical standard (p-coumaric acid, rutin, resveratrol, coumarin, liquiritigenin, quercetin, luteolin, and chrysin) at a concentration of 75 µg/mL was detected using UFLC–DAD–UV–Vis with a specific wavelength of 277 nm for flavones and flavanols; Supplementary Figure S4. The chromatogram of the analytical standard (artepillin C) at a concentration of 75 µg/mL was detected using UFLC–DAD–UV–Vis with a specific wavelength of 315 nm for hydroxycinnamic acid derivatives; Supplementary Figure S5. The chromatogram of the GBPUP extract (sample January 2021) at a concentration of 600 µg/mL was detected using UFLC–DAD–UV–Vis with a wavelength of 315 nm; Supplementary Table S1. Data on the period and apiaries of collection, total phenol, flavonoid, and flavanone content, and IC_50_ value for DPPH• free radical scavenging capacity; Datasets for multivariate statistical analysis (Circos plot and ASCA) (months of the year, apiaries, concentrations of each metabolite quantified during the seasonal study, MICs of the bacterial strains, IC_50_ of leishmanicidal activity) will be made available by the authors upon request. References [68,69,70] are cited in the Supplementary Materials.

## Nomenclature

The following nomenclatures according to the IUPAC are used in this manuscript: 2-coumaric acid, (E)-3-(2-hydroxyphenyl)prop-2-enoic acid; artepillin C, E)-3-[4-hydroxy-3,5-bis(3-methylbut-2-enyl)phenyl]prop-2-enoic acid; caffeic acid, 3,4-dihydroxycinnamic acid or (E)-3-(3,4-dihydroxyphenyl)prop-2-enoic acid; catechin, (2R,3S)-2-(3,4-dihydroxyphenyl)-3,4-dihydro-2H-chromene-3,5,7-triol; chrysin, 5,7-dihydroxy-2-phenylchromen-4-one; ferulic acid, (E)-3-(4-hydroxy-3-methoxyphenyl)prop-2-enoic acid; galangin, 3,5,7-trihydroxy-2-phenylchromen-4-one; kaempferol, 3,5,7-trihydroxy-2-(4-hydroxyphenyl)chromen-4-one; liquiritigenin, (2S)-7-hydroxy-2-(4-hydroxyphenyl)-2,3-dihydrochromen-4-one; luteolin, 2-(3,4-dihydroxyphenyl)-5,7-dihydroxychromen-4-one; naringenin, (2S)-5,7-dihydroxy-2-(4-hydroxyphenyl)-2,3-dihydrochromen-4-one; *p*-coumaric acid, (E)-3-(4-hydroxyphenyl)prop-2-enoic acid; pinobanksin, (2R,3R)-3,5,7-Trihydroxyflavanone or (2R,3R)-3,5,7-Trihydroxy-2-phenyl-2,3-dihydro-4H-chromen-4-one; pinocembrin, (2S)-5,7-dihydroxy-2-phenyl-2,3-dihydrochromen-4-one; pinostrobin, (2S)-5-hydroxy-7-methoxy-2-phenyl-2,3-dihydrochromen-4-one; quercetin, 2-(3,4-dihydroxyphenyl)-3,5,7-trihydroxychromen-4-one; resveratrol, 5-[(E)-2-(4-hydroxyphenyl)ethenyl]benzene-1,3-diol; rutin, 2-(3,4-dihydroxyphenyl)-5,7-dihydroxy-3-[(2S,3R,4S,5S,6R)-3,4,5-trihydroxy-6-[[(2R,3R,4R,5R,6S)-3,4,5-trihydroxy-6-methyloxan-2-yl]oxymethyl]oxan-2-yl]oxychromen-4-one.

## Abbreviations

The following abbreviations are used in this manuscript:

BOD	Biochemical Oxygen Demand
DPPH•	(2,2-diphenyl-1-picrylhydrazyl) radical
FBS	Fetal Bovine Serum
GBPUP	Greenish-Brown Propolis from União dos Palmares, Alagoas, Brazil
Ld3MST Enzyme	Ld3MST Enzyme, 3-Mercaptopyruvate Sulfurtransferase Enzyme
MHB	Mueller–Hinton broth
MIC	Minimum Inhibitory Concentrations
NNN	Novy–MacNeal–Nicolle medium
DIABLO	Data Integration Analysis for Biomarker discovery using Latent Components
ASCA	ANOVA Simultaneous Component Analysis
