# Supplementary material for: Seasonal Variation of a New Brazilian Greenish-Brown Propolis Type: Chemical Composition and Antioxidant, Antimicrobial, and Antileishmanial Activities
Source: Molecules. 2026 Apr 27;31(9):1447. doi: 10.3390/molecules31091447 (PMC13165146; doi:10.3390/molecules31091447)
Supplement: Supplementary file 1 [file molecules-31-01447-s001.zip › molecules-4182100-supplementary.pdf]

Section S1. Total Phenolic Content, Total Flavonoid Content, Total Flavanone Content, and Determination of IC_{50} Values in DPPH Scavenging Capacity Assay

Supplementary Table S1. Data on the period and apiaries of collection, total phenol, flavonoid, and flavanone content, and determination of IC_{50} values in DPPH• scavenging capacity assay.

Month of collection	Collection location during propolis seasonal study (sample origin)	Total phenolic content (mg GAE/g extract ± SD)	Total flavonoid content (mg QE/g Extract ± SD)	Total flavanone content (mg PE/20mg extract ± SD)	DPPH• scavenging capacity IC_{50} (μg/mL ± SD)
SEP/2020	Mata dos Frios	58,75 ± 4,71	31,92 ± 0,37	1,75 ± 0,18*	$^{\Phi}$77,63 ± 1,06
OCT/2020	Mata dos Frios	59,05 ± 5,53***	49,66 ± 0,86	1,44 ± 0,19	$^{\Phi}$44,65 ± 0,37
NOV/2020	Serra da Barriga	$^{\Phi}$68,3 ± 1,09	45,38 ± 2,42	1,33 ± 0,13	$^{\Phi}$40,24 ± 0,09
DEC/2020	Mata Microondas	$^{\Xi}$82,91 ± 7,14	57,89 ± 2,60	1,52 ± 0,16	$^{\Phi}$45,67 ± 0,39
JAN/2021	Mata dos Frios	97,36 ± 4,97**	$^{\lfloor;\xi;\Phi;\Xi;\Gamma}$66,54 ± 2,03	1,43 ± 0,16	$^{\xi}$33,81 ± 0,23
FEB/2021	Branquinha	88,88 ± 4,09***	46,84 ± 2,48	1,66 ± 0,19	$^{\Phi}$44,34 ± 0,24
MAR/2021	Branquinha	$^{\Phi}$; $^{\Xi}$37,42 ± 13,72***	$^{\Gamma}$28,23 ± 0,32	2,04 ± 0,24$^{\Psi}$	$^{\Phi}$51,63 ± 0,49
APR/2021	Mata dos Frios	$^{\Xi}$117,79 ± 4,25	55,33 ± 1,23	1,79 ± 0,21$^{\perp}$,	$^{\Phi*}$32,26 ± 0,14
MAY/2021	Mata dos Frios	$^{\Xi}$134,18 ± 12,41***	$^{\Xi}$42,91 ± 1,55	1,45 ± 0,17	$^{\Phi}$43,72 ± 0,12
JUN/2021	Mata dos Frios	81,27 ± 6,18***	$^{\Phi}$39,23 ± 3,57	1,15 ± 0,07*;**,$^{\Psi}$, $^{\perp}$	$^{\Phi}$37,82 ± 0,21
JUL/2021	Sueca	89,06 ± 17,54***	$^{\xi}$35,03 ± 1,87	1,66 ± 0,27	$^{\Phi}$42,11 ± 0,52
AUG/2021	Mata dos Frios	58,04 ± 3,24***	$^{\lfloor}$27,72 ± 7,16	1,90 ± 0,19**	$^{\Phi}$71,24 ± 0,20

Total phenolic content **[68]**: ANOVA one-way, Bonferroni's multiple-comparison test: ($P < 0.0001$, Bonferroni); May extract ***t value > 6.500; **t value = 5.316; march extract $^{\Phi}$t value = 4.446; $^{\Xi}$ t value > 6.500. Total flavonoid content **[68]**: ANOVA one-way, Bonferroni's multiple-comparison test: ($P < 0.0001$, Bonferroni); January extract $^{\Gamma}$t value = 6.409; $^{\Xi}$t value = 3.923; $^{\Phi}$t value = 4.511; $^{\xi}$t value = 5.233; $^{\lfloor}$t value = 6.382. Total flavanone content **[69]**: ANOVA one-way, Bonferroni's multiple-comparison test: ($P < 0.003$, Bonferroni); June extract *t value = 3.888; **t value = 4.837; $^{\perp}$t value = 4.151; $^{\Psi}$ value = 5,715. DPPH• Scavenging capacity IC_{50} **[70]**: ANOVA one-way, Bonferroni's multiple-comparison test: ($P < 0.0001$, Bonferroni); April extract $^{\Phi}$t value > 14.880; $^{\xi}$t value = 4.446.

Section S2

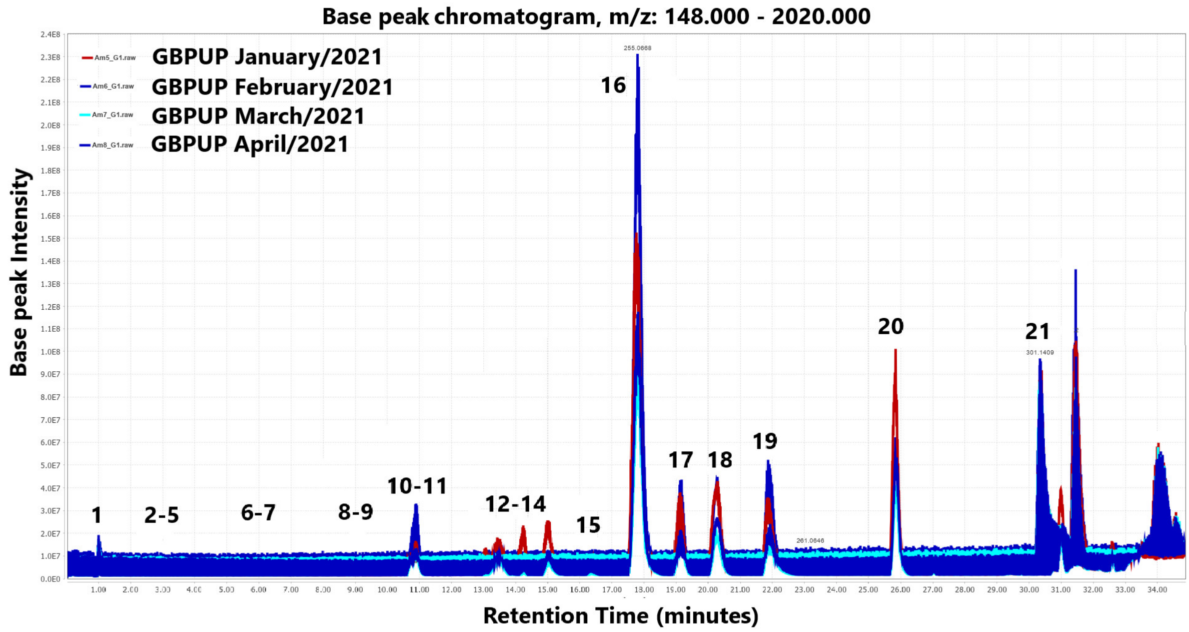

Supplementary Figure S1. Superposition of the chromatograms of the GBPUP seasonal extract samples for the months January 2021, February 2021, March 2021, and April 2021.

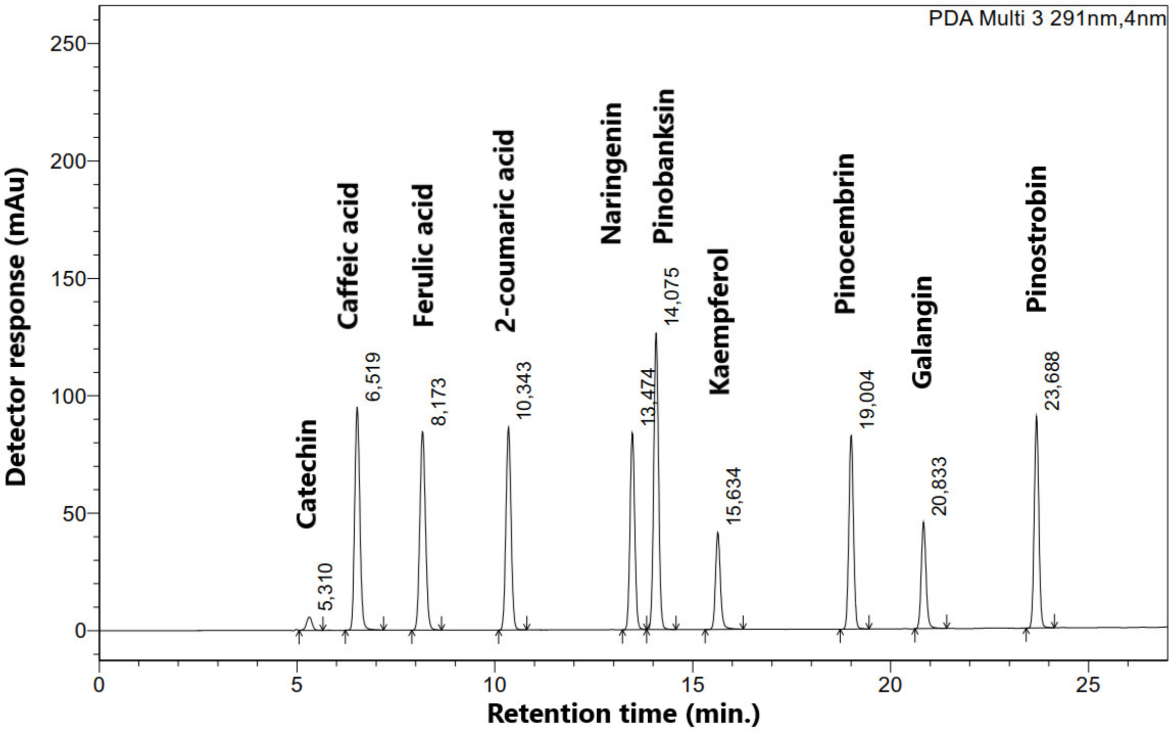

Supplementary Figure S2. The chromatogram of mixture 1 of the analytical standard at a concentration of 75 μg/mL was detected using UFLC–DAD–UV–Vis with a specific wavelength of 291 nm for flavanones.

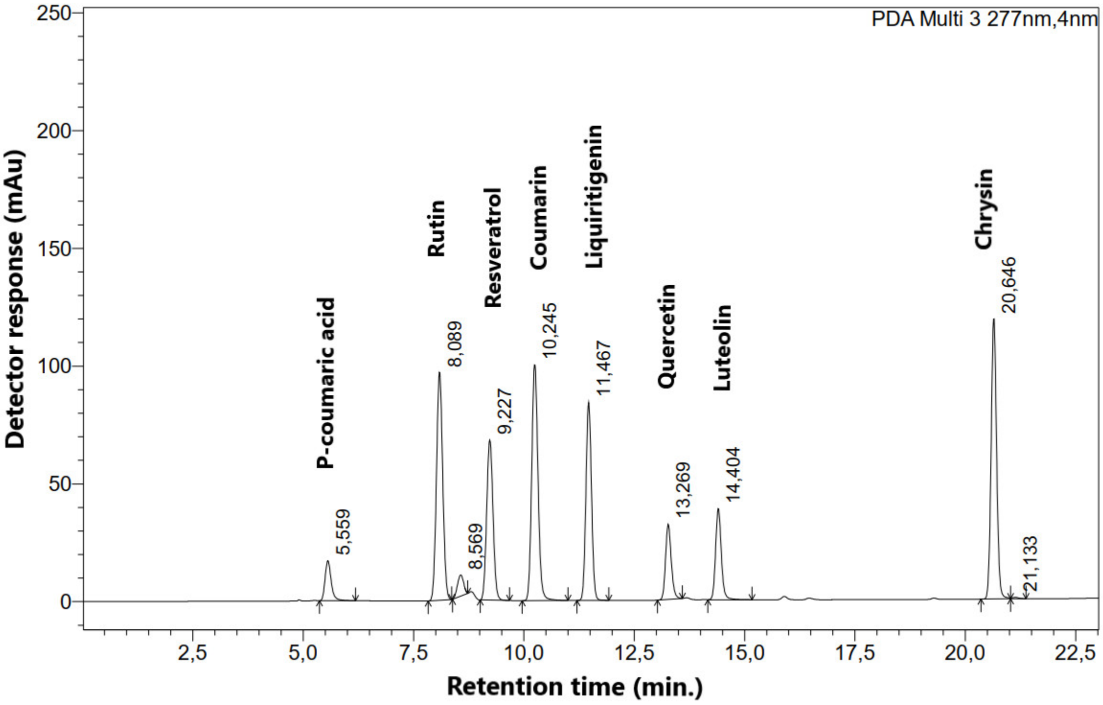

Supplementary Figure S3. The chromatogram of mixture 2 of the analytical standard (*p*-coumaric acid, rutin, resveratrol, coumarin, liquiritigenin, quercetin, luteolin, and chrysin) at a concentration of 75 μg/mL was detected using UFLC–DAD–UV–Vis with a specific wavelength of 277 nm for flavones and flavanols.

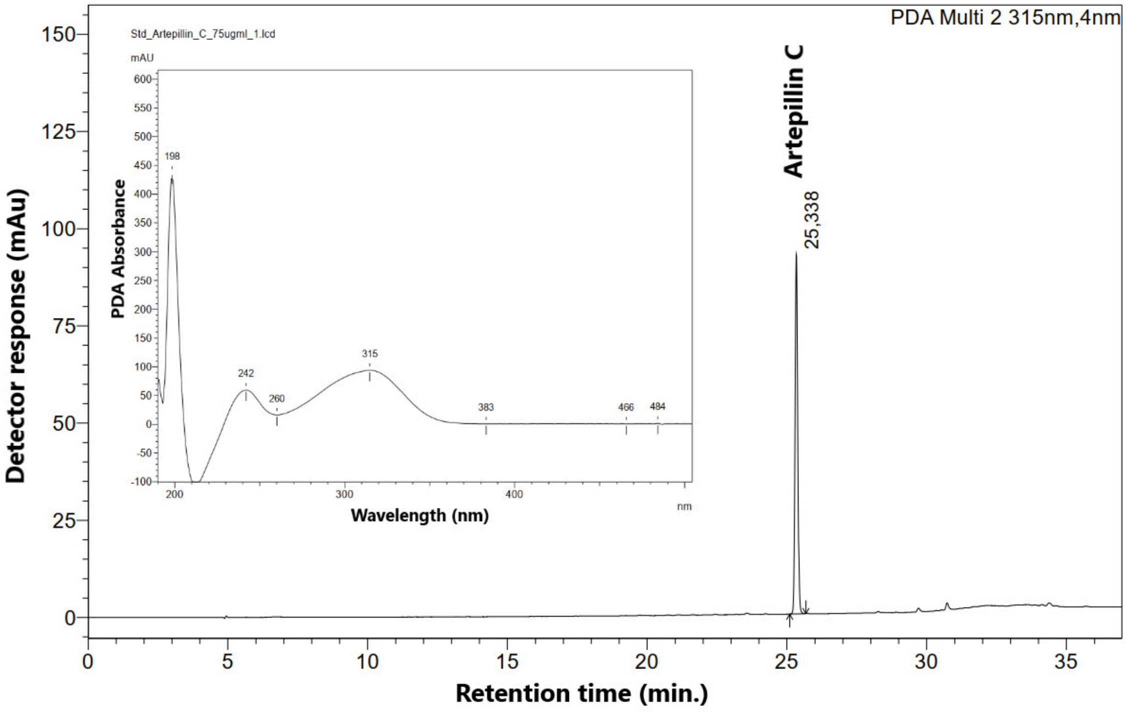

Supplementary Figure S4. The chromatogram of the analytical standard (artepillin C) at a concentration of 75 μg/mL was detected using UFLC–DAD–UV–Vis with a specific wavelength of 315 nm for hydroxycinnamic acid derivatives.

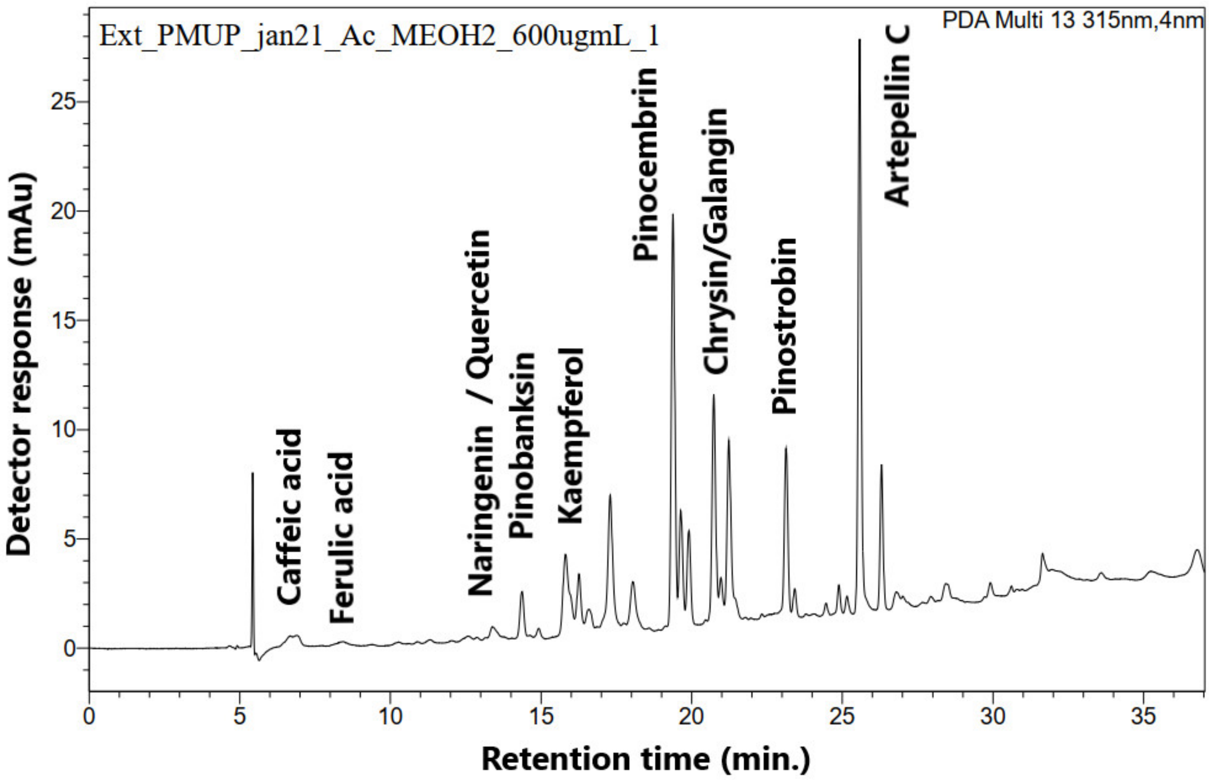

Supplementary Figure S5. The chromatogram of the GBPUP extract (sample January 2021) at a concentration of 600 μg/mL was detected using UFLC–DAD–UV–Vis with a wavelength of 315 nm.
